# Application of Ultraviolet Laser Working in Cold Ablation Conditions for Cutting Labels Used in Packaging in the Food Industry

**DOI:** 10.3390/ma13225245

**Published:** 2020-11-20

**Authors:** Łukasz Bohdal, Leon Kukiełka, Radosław Patyk, Rafał Gryglicki, Piotr Kasprzak

**Affiliations:** 1 Department of Mechanical Engineering, Koszalin University of Technology, Racławicka 15-17 Street, 75-620 Koszalin, Poland; leon.kukielka@tu.koszalin.pl (L.K.); radoslaw.patyk@tu.koszalin.pl (R.P.); 2 MATSIM Sp. z o.o., Zwycięstwa 28/3, 75-037 Koszalin, Poland; rafal.gryglicki@matsim.pl (R.G.); piotr.kasprzak@twohorses.pl (P.K.)

**Keywords:** UV laser, cold ablation, polypropylene multilayer foil, cutting, degraded zone, optimization, reverse task

## Abstract

This work presents experimental studies aiming at the development of new technology and guidelines for shaping labels from polypropylene multilayer foil using an ultraviolet (UV) laser cutting operation. Currently on production lines, the shaping of labels is undertaken by mechanical cutting or laser cutting, taking into account the phenomenon of hot ablation. These technologies cause many problems such as burr formation on labels sheared edges, rapid tool wear, or heat-affected zone (HAZ) formation. The experimental tests were carried out on a specially designed laser system for cutting polypropylene foil using the phenomenon of cold ablation. Parametric analyses were conducted for several foil thicknesses *t* = 50, 60, 70 and 80 µm. The process parameters were optimized in terms of high efficiency and high labels-cut surface quality. A new criterion has been developed for assessing the quality of UV laser cutting of polypropylene foils. The results indicate a significant effect of the cutting speed and laser frequency on the width of the degraded zone on the sheet cut edge. As a result of a developed optimization task and reverse task solution it is possible to cut labels at high speeds (*v* = 1.5 m/s) while maintaining a high quality of cut edge free of carbon, delamination and color changes. A degraded zone does not exceed in the examined cases *s* ≤ 0.17 mm.

## 1. Introduction

The IML (in-mold labeling) manufacturing process is usually performed by several steps. First, the automation system takes a single label from the appropriate label magazine, then the robot passes the label to an open injection mold suspended on an injection molding machine [1,2]. After the robot is withdrawn, the injection molding machine closes the injection mold and plastic injection is carried out which fills the mold space. The material is inseparably bonded to the label, creating a ready packaging. The injection molding machine opens the injection mold, and the finished packaging is removed from the mold. The whole process is repeated. The printed label is placed on the outer walls of the packaging and it is a carrier of visible information (product name, weight and composition, energy value, saturated fat, carbohydrate, sugar, protein and salt), advertising in graphic form, manufacturer’s logos and the country of origin of the food. In-mold labels can be found on cans, cups and lids available on supermarket shelves. Polypropylene or polystyrene is commonly used as label material, with typical thickness from 40 to 80 micrometers. They can be of many different sizes and shapes. They are printed on special substrate and the printed labels are die-cut and stacked. The die-cut process is carried out using components of die and punch assembly (in mass production). Tooling in this type of production is very expensive but the cost of these components is compensated for by the number of cut elements, which is counted in millions of pieces. For unit or small-lot production, cutting with the use of components of die and punch assembly is unprofitable, especially for large and complex label shapes (large punch and die with complex outlines is needed, which affects the cost of its manufacture). It is necessary to ensure the mechanical strength and stability during the die-cutting and molding process which is very difficult to work with during printing and die-cutting of labels due to them being very elastic and easily breakable.

Scrap generation is another problem on production lines. Mechanical cutting generates waste in the form of foil openings (space between the patterns is a few millimeters), which in mass production at the level of millions of pieces generates a significant amount of waste of foil. Cutting of materials with a composite structure, such as multilayer labels causes rapid wear of the cutting edges of the punch and die. It is also problematic to set the optimal clearance between the punch and die before cutting thin materials which influence during the process the formation of burrs and debris on the cut surface [3,4]. It has been found that in comparison with mechanical cutting, for example, blanking or guillotining, lasers cause less stress in the material and reduce cut surface defects, for example burrs and edge waves [5,6].

Recent developments introduce the laser-cutting option instead of the standard die-cut plates for various materials. Miraouri et al. [7] analyzed high-power CO_2_ laser cutting of steel plates. The effect of the input laser cutting parameters on the cut surface quality was analyzed. An overall optimization was applied to find out the optimal cutting setting that improves the cut surface quality. Caiazzo et al. [8] examined the application of the CO_2_ laser cutting process to three thermoplastic polymers—polyethylene, polypropylene, polycarbonate—in different thicknesses ranging from *t* = 2 to 10 mm. Eltawahni et al. [9] related the cutting edge quality parameters (responses), namely upper kerf, lower kerf, ratio of the upper kerf to lower kerf and cut edge roughness, to the process parameters considered in this research to find out the optimal cutting conditions of polyethylene. Work presented by Abrao et al. [10] evaluated the effect of the processing parameters on the quality of the cut for several engineering plastics. Uebel and Bliedtner [11] presented different cutting methods and types of laser which were used in the examinations in order to achieve a low-distortion and high-quality cutting of the materials in the sintered and unsintered state at high process velocities. The test results were analyzed with the use of picosecond lasers at different wavelengths (1064 nm, 532 nm and 355 nm). The tests with the sintered materials showed that process quality increases with shorter pulse duration. Additionally, the results can be improved with shorter wavelengths. Kukiełka et al. [12] analyzed the possibility of using fiber and diode lasers for the formation of workpieces from polypropylene multilayer foil using cutting technology.

Recently, ultraviolet (UV) lasers with ultra-short pulses have become popular in the nano-, femto- or picosecond range [13,14,15]. They are used in aeronautics, automotive, medical engineering, electronics. The specificity of the UV laser is the application the phenomenon of cold ablation. The process is adiabatic, and the cutting zone could be much smaller compared to other lasers. It is possible to obtain a reduced heat-affected zone (HAZ) that minimizes burring, charring, and other negative effects of thermal stress normally associated with higher-powered lasers. Typically UV lasers, are considered to be one of the ideal tools for precise micromachining due to less mechanical and thermal stress. A much shorter wavelength of UV lasers, compared to, for example, CO_2_ operating in the infrared, allows for precise focusing, and thus very high machining accuracy. The high energy density of radiation allows processing of different materials equally well, e.g., polymer laminates, ceramic substrates. Nowak et al. [16] made calculations of predicted values of the key process parameters for laser machining associated with cold laser ablation of ceramic materials in the green state. The values of threshold fluence, particle escape velocity and material removal rate were calculated. Witkowski et al. [17] analyzed the problem of the laser micromachining issues with cold ablation. In a study, the impact of the laser beam trajectory on the effect of interaction on matter during the laser milling process was investigated. Garasz et al. [18] performed the results of the experimental parametric study on quality, efficiency and accuracy of laser micromachining based on Yb:KYW subpicosecond laser and a Ti:S femtosecond laser. A comparison between the two methods was made. The results show that minimal HAZ can be obtained by shortening the duration of the laser pulse to the range of tens of femtoseconds. Mirza et al. [19] analyzed the cold ablation mechanisms separated in time based on the evolution of ablation craters of wide-bandgap transparent materials. Numerical simulations were performed to gain insight into the processes triggered by laser radiation in glass. According to results multi-pulse ablation at moderate laser fluences can lead to improvement of crater quality. Oosterbeek et al. [20] analyzed the cold ablation process during ultrafast laser micromachining. The laser ablation threshold fluence (minimum pulse energy density at which material removal is achieved) was measured for selected material. Rethfeld et al. [21] discussed modeling issues of ultrafast laser ablation of solids, as well as the underlying processes, on a broad range of timescales. Mathematical descriptions of cold ablation and laser absorption in metals, semiconductors and dielectrics were presented.

Labels must be clear which means that very little chemical change have to be obtained in the material and no delamination effect after process.

Some researchers analyzed the possibility of using UV lasers in a cutting process of various materials. Sotnikov et al. [22] analyzed the effect of elliptical beam shapes on cutting performance of silicon using a diode pumped solid state Q-switched UV laser operating at the wavelength of 355 nm. Zhang and Faghri [23] developed a theoretical model to predict the cavity formation for UV laser micro-machining with nanosecond pulse duration. Wang et al. [24] used a UV laser with 355 nm wave-length to cut printed circuit boards. In reference [25], the feasibility of employing a femtosecond laser for the rapid fabrication of micro-mechanical test structures, in the particular case of cantilevers in tungsten foils, with lengths up to several hundred micrometers, has been demonstrated. There are, however, challenges in laser processing of thin foils where the goal is to minimize HAZ and to use a high processing speed [26,27,28]. The main problem in practical industry is choosing the type of laser and the amount of adjustable laser cutting parameters and the fact that the influence of these parameters on the process is not fully understood [29,30,31,32]. This makes it difficult to control the process. In practice, the right setup for the laser parameters is mostly found by trial and error combined with experience.

**Figure 1:**
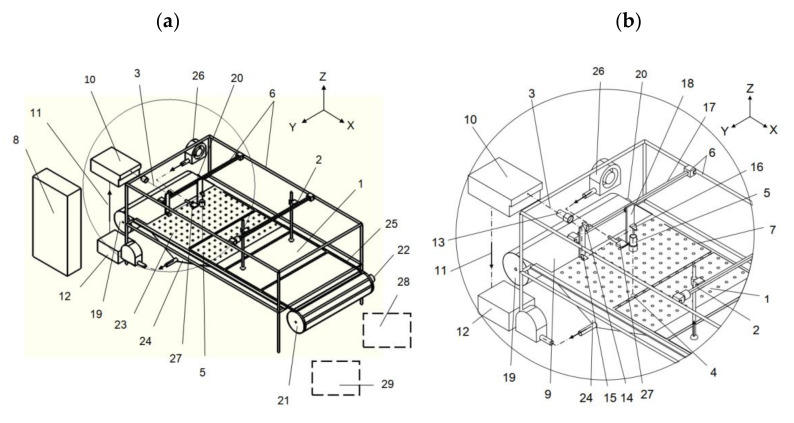
Diagram of the stand for cutting labels from a multilayer polypropylene film during production of in-mold labelling (IML) (**a**) and a view of a movable system based on a plotter for ultraviolet (UV) laser cutting (**b**): 1—label, 2—manipulator, 3—UV laser beam, 4—UV laser spot, 5—UV laser cutting head, 6—flatbed plotter, 7—label contour, 8—control cabinet, 9—foil, 10—laser source, 11—coolant, 12—cooler, 13—expander, 14—mirror I, 15—mirror II, 16—mirror III, 17—crossbeam, 18—handle, 19—foil unwinding roller, 20—unwinding roller drive, 21—openwork roll, 22—openwork roll drive synchronized with the drive of the unwinding roller, 23—perforated table, 24—vacuum chamber, 25—openwork, 26—dry air compressor, 27—nozzle for drying and cleaning the head, 28—robot feeding the film to the injection molding machine, 29—injection molding machine.

The aim of this paper is to establish and to assess UV laser processing as a novel technique for the fabrication of labels made from polypropylene multi-layer foil using IML technology. In Figure 1 the description of the laser system is given. Then the performance of the laser system for the fabrication of labels is assessed and process parameters are optimized in terms of high efficiency and cut surface quality. Finally, we answer the question of whether the developed laser system enables the mass-production of labels in the production process immediately before their insertion into the injection mold (IML).

The mobile system for cutting labels (1) shown in Figure 1 is placed in front of the manipulator (2), and the cutting process is carried out using an ultraviolet laser (3) with the following parameters: UV ray wavelength *λ* = 355 nm, pulse energy *E* = 5–13 µJ, laser power *P* = 5 W, frequency *f* = 20–150 kHz and pulse duration *τ* = 15–35 ns. The laser beam (3) is focused on the surface of the foil in the form of a spot (4), with a diameter of less than *d* = 20 µm, by means of a cutting head (5), which is coupled to a flatbed plotter (6). Movement of the cutting head (5) in the XY plane, following the outer contour (7) of the label (1), is forced by the means of the linear drive of the flatbed plotter (6) along the X axis and the Y axis so as to provide the required travel in the X axis direction and the Y axis direction. Cutting the label (1), the instantaneous frequency *f* of the laser beam (3) is determined by the control program installed on the computer located in the control cabinet (8), so that for a given thickness *t* of the cut foil (9) and the resultant speed v of the cutting head (5) movement, the optimal frequency *f* of the ultraviolet laser beam is determined (3) ensuring the occurrence, while cutting the label (1), of the phenomenon of cold ablation, at the same time high scanning speed *v*. The path (7) of the movement of the cutting head (5) depends on its movement along mutually perpendicular axes X and Y, which are synchronized according to the coordinate values determined by the control program and with a given speed *v* and a given acceleration. An economically justified, accurate cut of the label (1) with the required shape (7) is obtained. The ultraviolet laser beam (3) with a wavelength of *λ* = 355 nm and a variable frequency f depending on the scanning speed *v*, emitted horizontally along the X axis from a coaxial ultraviolet laser source (10), which cooled by (11) fed from the cooler (13), passes through the expander (14) and falls on the fully reflecting mirror (15), after which the reflected ray (3) is further transmitted in the direction of the Z axis to the next mirror (16), where it undergoes another complete reflection and changes its direction horizontally along the Y axis and it is sent to the next mirror (17), where it undergoes another complete reflection and changes its direction to vertical Z, and finally the laser beam (3), after passing through the cutting head (5), is focused to form a spot (4) on the cut propylene foil (9). The energy of the focused UV laser beam causes the photochemical phenomenon of cold ablation in the film cutting zone (9) without the influence of heat, thanks to which the processes of breaking the chemical bonds of foil macromolecules and the transition of the material from the solid to the gas phase take place. The expander (14) and the mirrors (15) and (16) are attached to the crossbeam (18) and move with it in the direction of the X axis. The mirror (17) and the cutting head (5), on the other hand, are attached to the holder (19) and this in turn is connected to the crossbeam (18). The mirror (17) and the cutting head (5) move with the holder (19) in the direction of the Y axis.

## 2. Experiment Procedure

### 2.1. Material Characteristics

The development of material characteristics was aimed at verifying the data declared by the manufacturer available in [33]. In the research was used a five-layer antistatic foil dedicated for IML technology supplied in rolls with a width of 1040 mm, length 5000 mm and thickness *t* = 50 (31.25 g/m^2^, density 0.62 g/cm^3^), 60 mass 35.6 g/m^2^, density 0.63 g/cm^3^, 70 (mass 55.4 g/m^2^, density 0.78 g/cm^3^) and 80 (mass 64.3 g/m^2^, density 0.83 g/cm^3^) micrometers. As part of the preliminary tests, surface mass, foil thickness, tensile strength and UV transmittance were verified. Based on the conducted tests, it was found that the results of the measurements of the indicated parameters of the foil are in accordance with the producer declaration and are within the tolerance.

**Figure 2:**
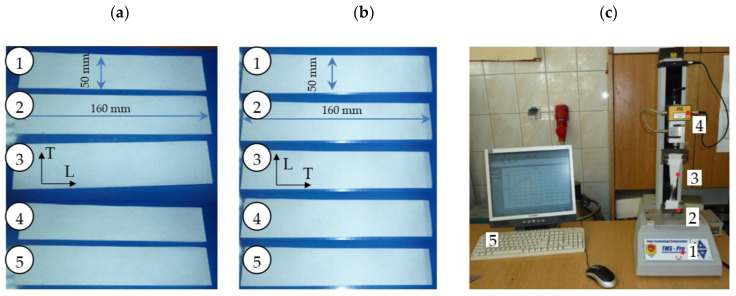
Photographs of samples for determining the tensile strength: (**a**) longitudinal direction, (**b**) transverse direction, (**c**) Mecmesin TMS PRO force and displacement station: 1—control panel, 2—clamping jaws, 3—sample, 4—FTC force sensor, 5—PC for recording and storing data.

Longitudinal and transverse tensile strength tests of analyzed polypropylene foil were carried out in accordance with the ASTM D882 (Standard test method for tensile properties of thin plastic sheeting) test method. According to the standard of test method, five samples with a length of *L* = 160 mm, measurement length *l* = 100 mm and width of *B* = 50 mm ± 0.5 mm were made for each test. Samples used for longitudinal and transverse tensile strength tests are shown in Figure 2a,b. The strength tests were carried out at the Mecmesin TMS PRO force and displacement test stand with an FTC force sensor with a measuring range of 0–2500 N and an accuracy of 0.1 N. The view of the strength test stand is shown in Figure 2c. The process of stretching the samples was carried out with speed *v* = 100 mm/min.

**Figure 3:**
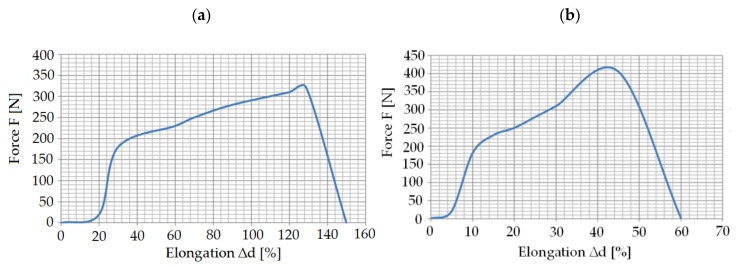
Graph of changes in tensile force as a function of elongation for a foil sample with a thickness of: (**a**) *t* = 80 µm, (**b**) *t* = 60 µm.

The results of longitudinal and transverse tensile strength tests are given in Table 1 and Table 2, Figure 3, respectively.

The tested polypropylene material exhibited a longitudinal tensile strength in the range of 75–82 MPa for all foil thicknesses and a transverse tensile strength in the range of 135–150 MPa. The longitudinal elongation was 125–135% and the transverse elongation was 40–45% for all foil thicknesses. These values are important in the production process when the foil is slightly tensioned during its development from the role. The values obtained are sufficient to avoid production defects resulting from excessive stretching of the foil or its breaking during unrolling.

### 2.2. Ultraviolet (UV) Laser Experimental Setup

**Figure 4:**
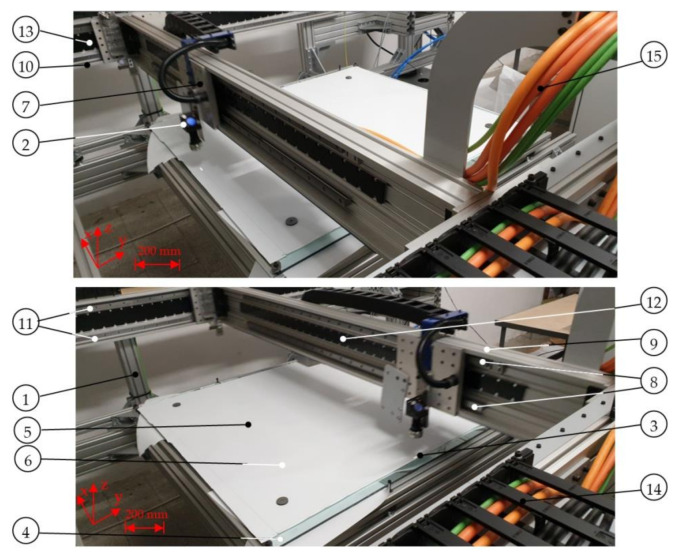
Test stand for cutting polypropylene foil using the phenomenon of cold ablation in various stages of operation: 1—frame made of aluminum profiles, 2—movable UV cutting head, 3—UV laser spot, 4—fixed table for laying polypropylene foil, unwound from a roll, 5—cut foil with dimensions 1040 mm × 1040 mm, 6—weights for pressing the foil against the table, 7—post for attaching the UV cutting head, moving along the X axis, 8—guide posts (7), 9—movable crossbar, 10—stationary upper right longitudinal member along the Y axis, 11—guides of the stationary upper right longitudinal member (10), 12—linear motor guide for driving along the X axis, 13—linear motor guide for driving along the Y axis, 14—chain guide for cables, 15—wires [34].

The experiments were carried out on the stand shown in Figure 4. The device frame is made of aluminum profiles. This allowed for a significant reduction in the cost of manufacturing the model and an increase in its flexibility. Therefore, it was possible to introduce changes and modifications to the structure easily. The workspace uses a fixed table for laying polypropylene foil, unwound from a roll. The table is attached to the guides. The foil is pressed against the ground by gravity. There is also a possibility of sticking the foil with double-sided tape or holding it with weights. To produce the laser beam, a stationary source was used along the upper right longitudinal member on the right side of the Y axis motor, outside the range of movement of the cutting head. The cutting head is movable. It has the ability to move in two directions, along the X and Y axes. A gantry system based on three linear motors was used to drive the head. Two synchronized linear motors are responsible for the movement in the Y axis. A movable upper crossbar is attached to these engines with a third linear motor attached and a UV cutting head holder. Thanks to this, it is possible to cut foil of any shape, in a maximum area of not less than 1040 mm × 1040 mm. The position of the head in the working space is read by means of a ruler along the X axis and synchronized two rulers attached to the upper right and left longitudinal members, in the area of operation of linear motors, which is designed to provide high system dynamics that are necessary to achieve short cycle times. Linear motors also do not require the application of power transmission assemblies and introduce smaller vibrations into the system. The laser beam incident along the Y axis guide is reflected by means of a mirror placed at the end of the X axis beam and directed along the X axis to the next mirror placed above the cutting head. This mirror directs the laser beam to the cutting head.

### 2.3. Experimental Research

Based on the analysis of the process, a set of factors significantly affecting the results factors was determined. Then, the ranges of variability of the examined (control) factors were determined. The tested factors include cutting speed in the range: *v* = *x*_1_ = 0.003–1.5 m/s and UV laser frequency in the range: *f* = *x*_2_ = 20–150 kHz. Tests are carried out according to the five-level uniform rotatable test plan (Table 3) [35], while statistical calculations were performed in accordance with the methodology described in [36,37].

**Figure 5:**
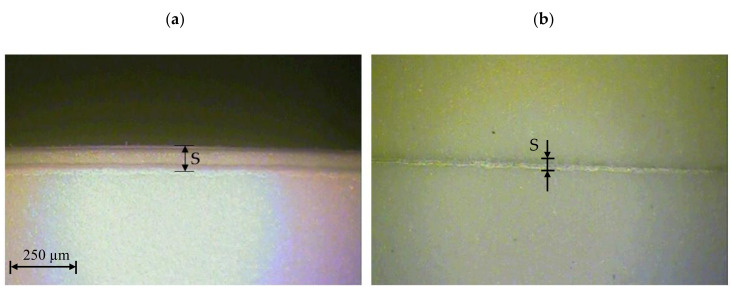
View of the cut surface of foil with thickness *t* = 50 µm: (**a**) *v* = 0.222 m/s, *f* = 39 kHz, (**b**) *v* = 0.751 m/s, *f* = 20 kHz.

The tests were carried out for four foil thicknesses: *t* = 50, 60, 70, 80 µm. For each thickness, the cutting parameters were changed as planned using three times the test repeatability for each plan level. The width of the degraded zone *S* (Figure 5) for each sample was measured at randomly selected points along the cutting line. The measurement of the width of the degraded zone and observation of the state of the intersection of the samples were made on a ND 1300 Quadra-Chek measuring microscope. The results obtained were averaged for each replicate. The following qualitative features were analyzed on each sample: carbon deposit (height and width), delaminated zone (delamination), and separated area of color changes. Because on each of the tested samples no such features were found, however, a zone with changed physical properties in relation to the native material was found; this zone was named as a degraded zone *S* and further tests were carried out for such a quality feature.

### 2.4. Experimental Results

**Figure 6:**
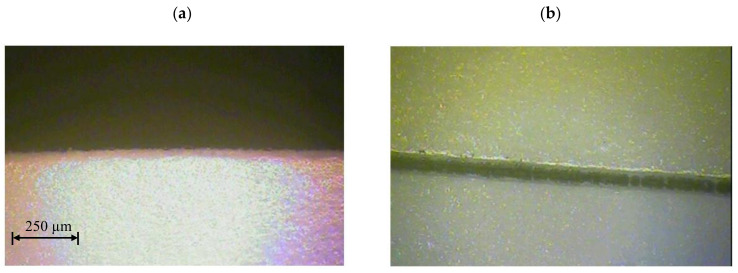
View of the cut surface of foil with thickness *t* = 60 µm: (**a**) *v* = 0.222 m/s, *f* = 39 kHz, (**b**) *v* = 1.28 m/s, *f* = 130 kHz.

**Figure 7:**
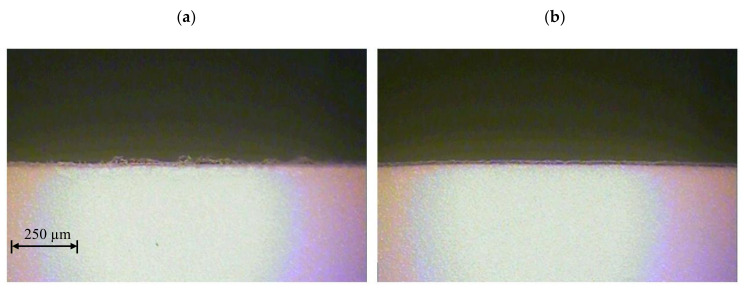
View of the cut surface of foil with thickness *t* = 70 µm: (**a**) *v* = 0.222 m/s, *f* = 39 kHz, (**b**) *v* = 1.28 m/s, *f* = 39 kHz.

**Figure 8:**
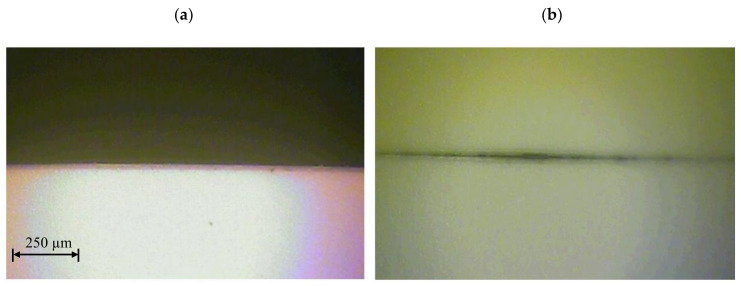
View of the cut surface of foil with thickness *t* = 80 µm: (**a**) *v* = 1.5 m/s, *f* = 85 kHz, (**b**) *v* = 0.751 m/s, *f* = 150 kHz.

**Figure 9:**
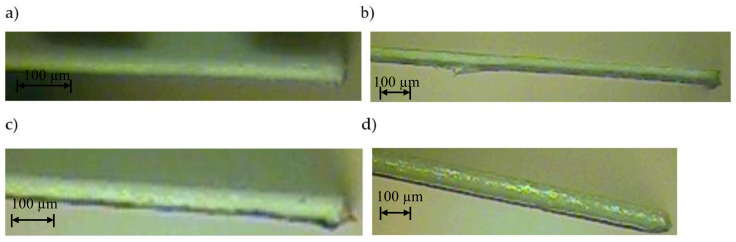
View of the cut surface of foil with thickness: (**a**) *t* = 50 µm, (**b**) *t* = 60 µm, (**c**) *t* = 70 µm, (**d**) *t* = 80 µm (*v* = 0.751 m/s, *f* = 85 kHz).

**Figure 10:**
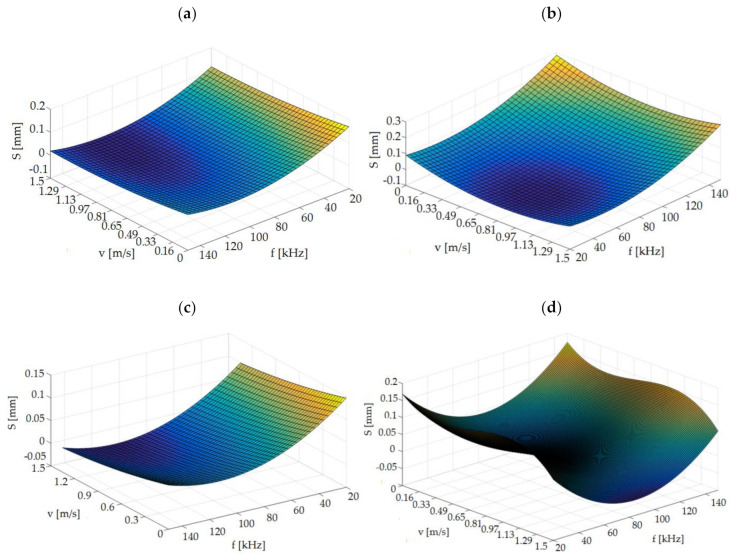
Graph of dependence *S* = *f*(*v*, *f*) for: (**a**) *t* = 50 µm, (**b**) *t* = 60 µm, (**c**) *t* = 70 µm, (**d**) *t* = 80 µm.

The results of the influence of cutting speed and UV laser frequency on the width of the degraded material zone for analyzed foil thicknesses are presented in Figure 5, Figure 6, Figure 7, Figure 8, Figure 9 and Figure 10. Exemplary photos of the cut surface of the foil with a thickness of *t* = 50 µm are shown in Figure 5. In this case it was not possible to achieve complete material separation only at a cutting speed of *v* = 0.751 m/s and UV laser frequency *f* = 20 kHz. For the rest of the cases, complete separation was obtained, and the obtained cutting edge was free of defects, e.g., slivers, edge waves and burrs.

Exemplary photos of the cut surface of the foil with a thickness of *t* = 60 µm are shown in Figure 6. Obtaining complete separation of the material was not possible with a cutting speed of *v* = 0.222 m/s and UV laser frequency *f* = 130 kHz. For this frequency value, it was also impossible to cut the material at a speed of *v* = 1.28 m/s (Figure 6b). It is also disadvantageous to use for this foil thickness parameters of *v* = 0.751 m/s and *f* = 150 kHz. In this case, the gap is narrow and there is no complete separation of the material.

Exemplary photos of the cutting surface of the foil with a thickness of *t* = 70 µm are shown in Figure 7. A complete separation of the material was obtained for all analyzed cases. For the cutting speed of *v* = 0.222 m/s, there are visible defects of the cut surface in the form of slivers, fines and debris (Figure 7a). Exemplary photos of the cutting surface of the foil with a thickness of *t* = 80 µm are shown in Figure 8. Complete separation was not obtained for cases of cutting at *v* = 0.751 m/s and UV laser frequency *f* = 20 kHz and *f* = 150 kHz. In other cases no defects of the cut edge were observed.

The next stage of research was the analysis of the cut surface of samples for possible occurrence of delamination effect. For this purpose, observations were made of the cut surface in the cross-section of the samples. Example of photos are presented in Figure 9. For all analyzed foil thicknesses and process parameters, no defects in the cut edge were observed in the form of delamination, excessive thickening or burr occurrence.

Detailed results of measurements of the width of degraded zone (*S*) for each plan level, depending on the given foil thickness, are presented in the Table 4, Table 5, Table 6 and Table 7.

The influence of analyzed parameters on width of degraded zone (*S*) is given on Figure 10. The test results show a significant influence of the tested technological parameters on the width of the degraded zone *S*. The influence of cutting speed *v* and source frequency *f* is strongly dependent on the thickness of the foil being cut. Therefore, it is impossible to use the same parameter settings for each type of foil thickness. For thicknesses of *t* = 50 µm and *t* = 70 µm, it is most advantageous to use source frequency above value *f* = 80 kHz. For the thickness of *t* = 60 µm, the smallest degraded zone can be obtained for a source frequency below *f* = 85 kHz in the tested speed range. For thicknesses of *t* = 80 µm, it is preferable to use values in the range of *f* = 60–90 kHz and high cutting speeds.

## 3. Process Optimization

An important problem in practical applications is the control of product performance (maintaining appropriate tolerances of the product shape, minimizing burrs and width of the degraded zone) already at the stage of its manufacture. Therefore, it is necessary to determine the optimal conditions for the implementation of the process. In the case of process optimization, optimal control factor settings are usually sought (in the case of the tested UV laser it is the cutting speed *v* and UV laser frequency *f*), for which the developed objective function obtains the extreme (minimum or maximum).

In the analyzed case, four types of regression function of the second kind constituting the mathematical model of the examined object were developed in the identification process. Each of the individual functions developed is only relevant for a given foil thickness (*t* = 50; 60; 70; 80 µm) and for the device tested. The research also found that the best feature describing the technological quality of cut labels from polypropylene foil would be the width of the degraded zone. One common assessment criterion was adopted because no such features were found such as maximum height and width of carbon deposit (if any), maximum width of the detached layer (if any), maximum separated area of foil color changes (if any), other potential deformations; at the same time, stating that there is a zone with altered physical characteristics compared to the characteristics of the native material. The developed type II regression functions in the case of process optimization can be objective functions for which minima should be found and optimal control parameters should be determined (cutting speed *v* and UV laser frequency *f*).

**Figure 11:**
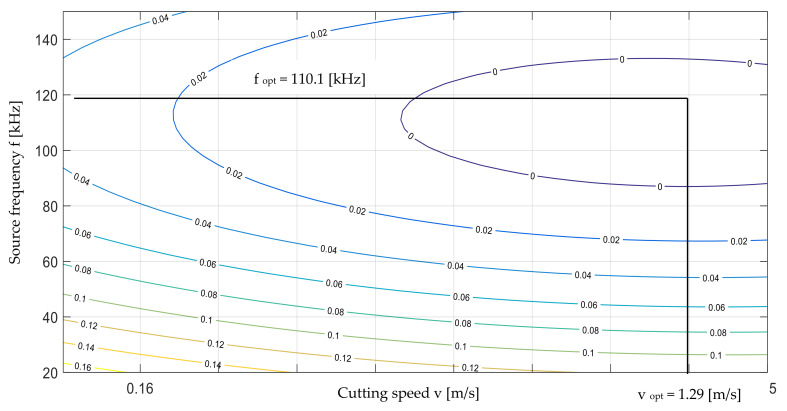
Dependence graph for polypropylene foil thickness *t* = 50 µm together with optimal solution marked (solution obtained by two methods).

In the case under consideration, a graphical method was used that allows an approximate solution to the problem, but it can be a simple method of verifying the exact solution obtained using the second gradient method. Optimization of the gradient cutting process was carried out using the Matlab program using the Optimization toolbox (Figure 11). Limitations imposed on controllable variables (cutting speed *v* and UV laser frequency *f* were determined for the considered process. The optimization task was formulated as follows:

Optimization given for each foil thickness *t* = 50; 60; 70 and 80 µm

*S* = *f*(*v*, *f*) → min

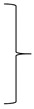

Degraded zone *S* → minimum0.003 ≤ *v* ≤ 1.5 m/sTechnological range for cutting speed *v*20 ≤ *f* ≤ 150 kHzTechnological range for the UV laser frequency *f*

As a result of static optimization, optimal values of decision variables were obtained, which are summarized in Table 8. The values of the parameters listed in the table should ensure that the degraded zone on cut surface of the label has a minimal width after the process.

## 4. Formulation of Reverse Task

Due to the fact that during the process of cutting labels using a UV laser it is not technically possible to constantly maintain optimal technological parameters (especially when this applies to cutting speed *v*), a function should be developed that allows the appropriate selection of the source frequency f depending on the obtained cutting speed *v*. This situation occurs when cutting small labels and when cutting corners (then the instantaneous speed is *v* = 0). For this problem, the following task was developed for each foil thickness *t* = 50; 60; 70 and 80 µm separately:

*f* = *f*(*v*) ?

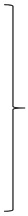


*What is the relationship between the frequency f of the UV laser beam and*

*the cutting speed v?*


**Assuming that:**
*S = f*(*v*, *f*) → minDegraded zone *S* → minimum0.003 ≤ *v* ≤ 1.5 [m/s]Technological range for cutting speed *v*20 ≤ *f* ≤ 150 [kHz]Technological range for the UV laser frequency *f*

**Figure 12:**
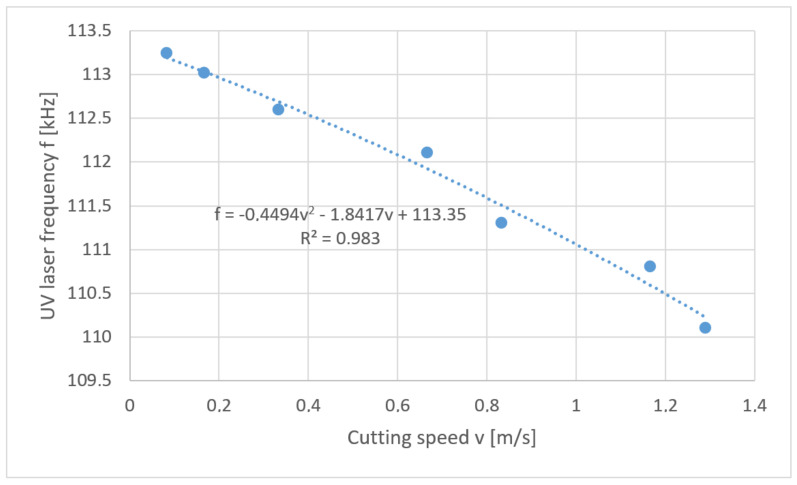
Reverse task solution for cutting foil with thickness *t* = 50 µm.

**Figure 13:**
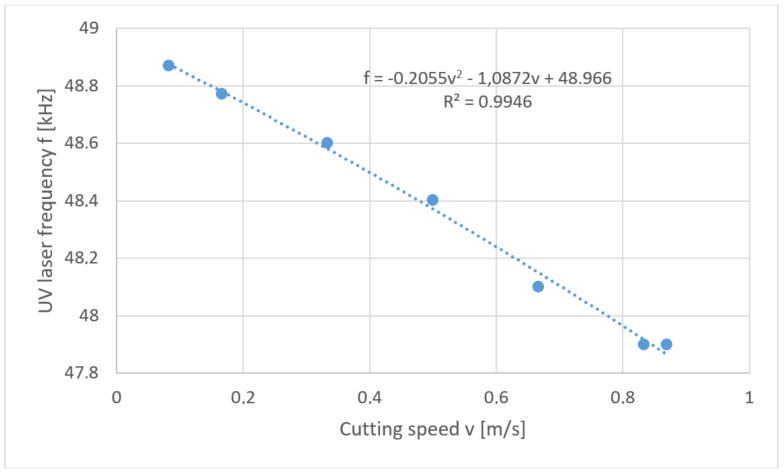
Reverse task solution for cutting foil with thickness *t* = 60 µm.

**Figure 14:**
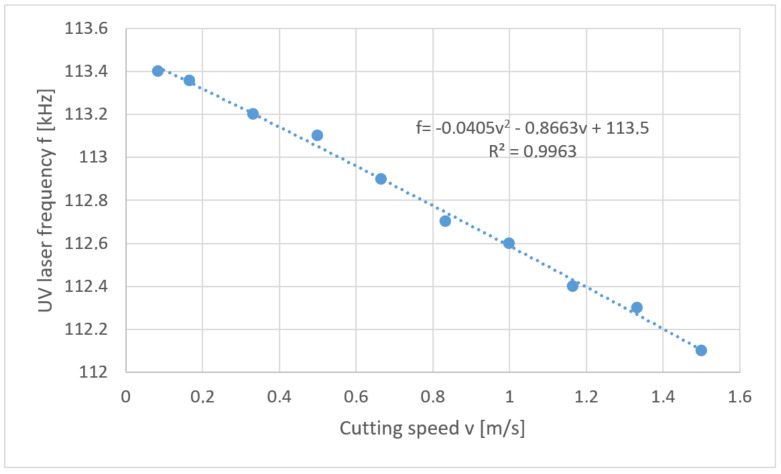
Reverse task solution for cutting foil with thickness *t* = 70 µm.

**Figure 15:**
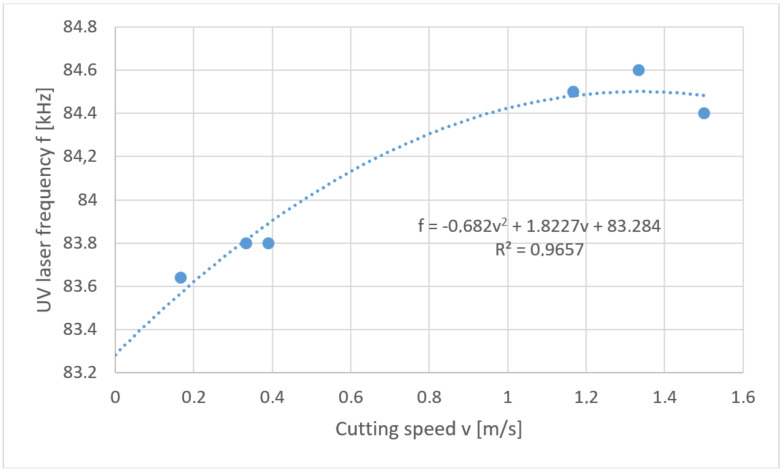
Reverse task solution for cutting foil with thickness *t* = 80 µm.

This problem is mathematically the reverse task. In the considered case a solution in the form of “soft” optimization was used. Below are the solutions to the problem posed in the form of a developed function of the relation *f* = *f* (*v*) for each thickness of polypropylene foil separately (Figure 12, Figure 13, Figure 14 and Figure 15).

The developed functions (reverse task solution) allow better control of the UV laser cutting process of polypropylene foils used for production of labels. The implementation of the developed functions into the laser device allows obtaining the best (possible in given process conditions) results—the minimum width of the degraded zone regardless of the cutting speed used. These solutions are particularly important when cutting out sharp corners, where low cutting speeds are obtained due to the limitation of the device (high overloads).

## 5. Experimental Research for Optimal Values of Decision Variables

**Figure 16:**
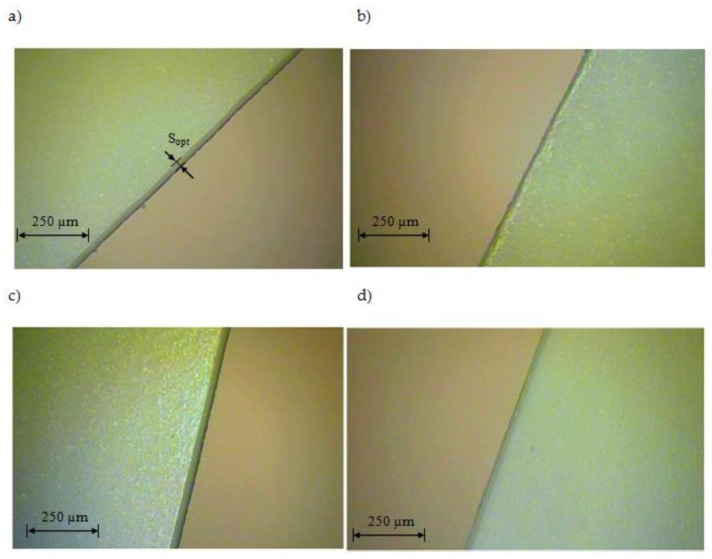
View of the cut surface of foil with thickness: (**a**) *t* = 50 µm (*v* = 1.29 m/s, *f* = 110.1 kHz), (**b**) *t* = 60 µm (*v* = 0.867 m/s, *f* = 47.9 kHz), (**c**) *t* = 70 µm (*v* = 1.499 m/s, *f* = 112.1 kHz), (**d**) *t* = 80 µm (*v* = 1.5 m/s, *f* = 84.8 kHz).

**Figure 17:**
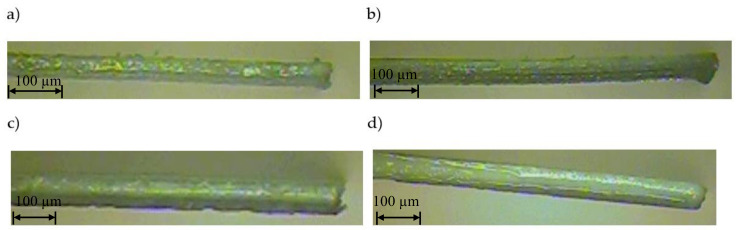
View of the cut surface in cross-section of the foil: (**a**) *t* = 50 µm, (**b**) *t* = 60 µm, (**c**) *t* = 70 µm, (**d**) *t* = 80 µm.

In the next stage of testing, tests were conducted for each foil thickness using optimal process parameters, i.e., UV laser frequency and cutting speed values (Table 8). The research was carried out for two replications. The view of cut surfaces of selected samples is given on Figure 16. The view of cut surfaces in cross section is given on Figure 17.

**Figure 18:**
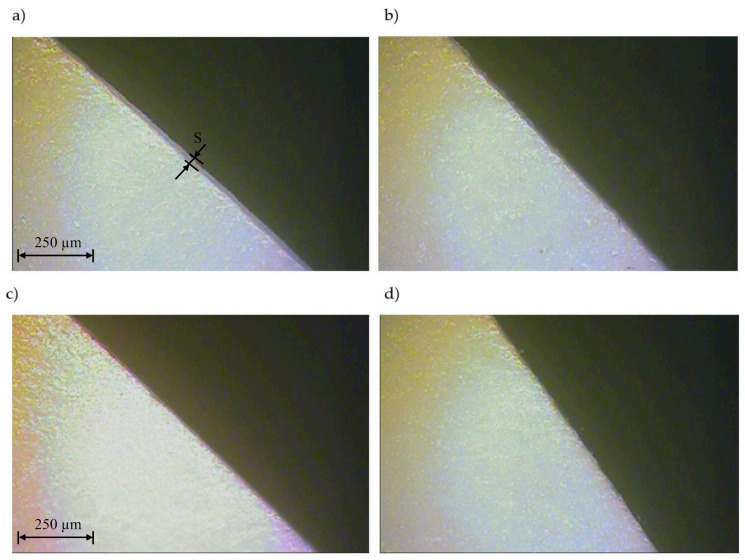
View of cut surface of foil thicknesses: (**a**) *t* = 50 µm, (**b**) *t* = 60 µm, (**c**) *t* = 70 µm, (**d**) *t* = 80 µm.

In the last stage of the research we performed the UV laser cutting process of polypropylene foil using a cutting speed of *v* = 1.5 m/s. The research was undertaken for optimal frequencies *f* selected for each PP foil thickness individually, i.e., for *t* = 50 µm, *f* = 110 kHz, for *t* = 60 µm, *f* = 47.9 kHz, for *t* = 70 µm, *f* = 112.2 [kHz], and for *t* = 80 µm, *f* = 84.5 kHz. Such a large frequency variation results from the properties of the material being cut and the characteristics of the laser source. The aim was to achieve maximum process efficiency. For the proper control of the laser, we used the function developed after solving the inverse task function *f* = *f* (*v*). The appearance of the intersection of samples is shown in Figure 18. The quality of the cut edge is very high and free from defects. The value of the *S* zone did not exceed the allowable *S* = 0.17 mm.

## 6. Conclusions

The aim of this paper was to establish and assess UV laser processing as a novel technique for the fabrication of labels made from polypropylene multilayer foil by IML technology. For this purpose, the laser system was built. Next, it was determined how individual process settings affect the technological quality of the IML label and whether and what interaction occurs between individual process settings. Development of the relationship between cutting parameters and product quality as well as process efficiency and energy consumption allow for forecasting the product quality after the process for given process implementation conditions or determining the required cutting process implementation conditions in terms of the required product quality.

Based on the conducted research, it can be stated that:

- Due to the fact that there are no qualitative features, i.e., carbon deposit, detached zone, and zone of color changes in foils, a new quality criterion has been developed for assessing the quality of UV laser cutting of polypropylene foils. A zone with changed physical properties (e.g., different edge strength) was found compared to the native material, and therefore this area was called a degraded zone and further tests were carried out for such a quality feature.
- In each of the examined cases (for samples after optimization) no exceedances of the size of defects of the cut labels were found, which would eliminate the developed method from industrial applications, as no:
  — changes in the thickness of the material being cut (adversely if the thickness increases—this may occur in the event of carbon deposits or foil delamination),
  — the width of the discoloration zone coincides with the width of the degraded zone and its value does not exceed in the examined cases of acceptable value *s* ≤ 0.17 mm.
- Developed mathematical models (different for each foil thickness *t*) were optimized by two methods: the graphic method and gradient method. The results obtained are presented in common charts while obtaining the compliance of solutions.
- In addition, for technological reasons it was necessary to develop and solve an inverse task that allows obtaining optimal values of the width of the degraded zone S by changing the source frequency *f* as a function of the set cutting speed (function *f* = *f* (*v*)).
- The optimization process followed by the solution of the reverse task for each case of foil thickness separately allows the process to be controlled (in terms of efficiency, energy consumption) and at the same time high-quality end products to be obtained.
- Implementing control of the laser device models of source frequency changes as a function of the set cutting speed (function *f* = *f* (*v*)) allows optimal values of the width of the degraded zone S to be obtained.
- The obtained results confirm that the developed research methodology is correct and can be the basis for further research on the process of cutting the UV laser of other materials.
- Further studies of the laser cutting of foil with a UV laser in real conditions with high cutting speeds and acceleration for sheets with minimum dimensions of 1 × 1 m are planned on the modified settings of technological parameters (for example: focus and size of the spot, mirror- to-material distance) in order to increase the efficiency of the process.

## Figures and Tables

**Table 1:** Longitudinal tensile strength test results.

Sample No.	Sample Thickness *t* [µm]	Tensile Strength (Breaking Force F_max_) F [N]	Initial Measurement Length of the Sample l [mm]	Elongation Δd [%]	Breaking Engineering Stress σ [MPa]
1.	50	187.5	100	125	75
2.	60	234	100	128	78
3.	70	287	100	135	82
4.	80	320	100	129	80

**Table 2:** Results of transverse tensile strength tests.

Sample No.	Sample Thickness *t* [µm]	Tensile Strength (Breaking Force F_max_) F [N]	Initial Measurement Length of the Sample l [mm]	Elongation Δd [%]	Breaking Engineering Stress σ [MPa]
1.	50	350	100	40	140
2.	60	405	100	45	135
3.	70	525	100	43	150
4.	80	596	100	44	149

**Table 3:** Five-level rotatable plan of experiment.

Plan Level	Coded Variables		Real Variables	
	${\overset{ˇ}{\bar{x}}}_{1}$	${\overset{ˇ}{\bar{x}}}_{2}$	*v* [m/s]	*f* [kHz]
1	−	−	0.222	39
2	+	−	1.280	39
3	−	+	0.222	130
4	+	+	1.280	130
5	+α = 1.414	0	1.500	85
6	−α = −1.414	0	0.003	85
7	0	+α = 1.414	0.751	150
8	0	−α = −1.414	0.751	20
9	0	0	0.751	85
10	0	0	0.751	85
11	0	0	0.751	85
12	0	0	0.751	85
13	0	0	0.751	85

**Table 4:** The matrix of the experiment plan according to the five-level plan with the results for the film thickness *t* = 50 µm.

Plan Level	Real Variables		The Width of the Degraded Zone *S*			
	*v*	*f*	Average	Repeats		
				1	2	3
	[m/s]	[kHz]	[mm]	[mm]	[mm]	[mm]
1	0.222	39	0.03	0.029	0.03	0.031
2	1.280	39	0.027	0.025	0.027	0.029
3	0.222	130	0.0075	0.0065	0.0073	0.0087
4	1.280	130	0.0125	0.0103	0.0127	0.0145
5	1.500	85	0.01	0.005	0.01	0.015
6	0.003	85	0.1	0.05	0.1	0.15
7	0.751	150	0.0125	0.0115	0.0125	0.0135
8	0.751	20	0.2	0.15	0.2	0.25
9	0.751	85	0.01	0.005	0.01	0.015
10	0.751	85	0.01	0.009	0.01	0.011
11	0.751	85	0.01	0.007	0.01	0.013
12	0.751	85	0.01	0.005	0.012	0.013
13	0.751	85	0.01	0.005	0.006	0.019

**Table 5:** The matrix of the experiment plan according to the five-level plan with the results for the film thickness *t* = 60 µm.

Plan Level	Real Variables		The Width of the Degraded Zone *S*			
	*v*	*f*	Average	Repeats		
				1	2	3
	[m/s]	[kHz]	[mm]	[mm]	[mm]	[mm]
1	0.222	39	0.015	0.014	0.015	0.017
2	1.280	39	0.01	0.007	0.009	0.015
3	0.222	130	0.2	0.15	0.2	0.25
4	1.280	130	0.2	0.15	0.2	0.25
5	1.500	85	0.02	0.02	0.022	0.023
6	0.003	85	0.1	0.1	0.12	0.13
7	0.751	150	0.14	0.21	0.27	0.29
8	0.751	20	0.01	0.01	0.015	0.019
9	0.751	85	0.0075	0.0073	0.0078	0.0086
10	0.751	85	0.0075	0.0072	0.0075	0.0082
11	0.751	85	0.0075	0.0072	0.0075	0.0077
12	0.751	85	0.0075	0.0071	0.0075	0.0078
13	0.751	85	0.0075	0.007	0.0075	0.0085

**Table 6:** The matrix of the experiment plan according to the five-level plan with the results for the film thickness *t* = 70 µm.

Plan Level	Real Variables		The Width of the Degraded Zone *S*			
	*v*	*f*	Average	Repeats		
				1	2	3
	[m/s]	[kHz]	[mm]	[mm]	[mm]	[mm]
1	0.222	39	0.0075	0.007	0.0075	0.0085
2	1.280	39	0.007567	0.0075	0.0077	0.0085
3	0.222	130	0.005	0.005	0.0055	0.0058
4	1.280	130	0.0075	0.007	0.0075	0.0079
5	1.500	85	0.0067	0.001	0.0015	0.005
6	0.003	85	0.1	0.08	0.1	0.13
7	0.751	150	0.0075	0.0073	0.0075	0.0079
8	0.751	20	0.2	0.21	0.22	0.23
9	0.751	85	0.0125	0.0123	0.0125	0.0128
10	0.751	85	0.0125	0.0123	0.0125	0.0127
11	0.751	85	0.010833	0.01	0.013	0.016
12	0.751	85	0.01	0.01	0.011	0.012
13	0.751	85	0.01	0.01	0.011	0.013

**Table 7:** The matrix of the experiment plan according to the five-level plan with the results for the film thickness *t* = 80 µm.

Plan Level	Real Variables		The Width of the Degraded Zone *S*			
	*v*	*f*	Average	Repeats		
				1	2	3
	[m/s]	[kHz]	[mm]	[mm]	[mm]	[mm]
1	0.222	39	0.005	0.0045	0.005	0.0055
2	1.280	39	0.007567	0.0072	0.0075	0.0077
3	0.222	130	0.01	0.01	0.011	0.013
4	1.280	130	0.0075	0.007	0.0075	0.0083
5	1.500	85	0.005	0.005	0.0055	0.0059
6	0.003	85	0.1	0.08	0.11	0.13
7	0.751	150	0.2	0.21	0.22	0.23
8	0.751	20	0.2	0.21	0.22	0.23
9	0.751	85	0.01	0.01	0.012	0.013
10	0.751	85	0.01	0.01	0.011	0.013
11	0.751	85	0.01	0.01	0.014	0.016
12	0.751	85	0.01	0.01	0.012	0.014
13	0.751	85	0.01	0.01	0.011	0.013

**Table 8:** Optimal values of decision variables.

Foil Thickness *t* [µm]	*v*_opt._ [m/s]	*f*_opt_ [kHz]	*S*_opt_ [mm]
50	1.29	110.1	0.0091
60	0.867	47.9	0.0479
70	1.499	112.1	0.1121
80	1.5	84.8	0.0848
